# Neuroimmune mechanisms in fear and panic pathophysiology

**DOI:** 10.3389/fpsyt.2022.1015349

**Published:** 2022-11-29

**Authors:** Katherine M. J. McMurray, Renu Sah

**Affiliations:** ^1^Department of Pharmacology and Systems Physiology, University of Cincinnati, Cincinnati, OH, United States; ^2^Veterans Affairs Medical Center, Cincinnati, OH, United States

**Keywords:** panic, fear, neuroimmune, panic disorder, interoception, body-to-brain

## Abstract

Panic disorder (PD) is unique among anxiety disorders in that the emotional symptoms (e.g., fear and anxiety) associated with panic are strongly linked to body sensations indicative of threats to physiological homeostasis. For example, panic attacks often present with feelings of suffocation that evoke hyperventilation, breathlessness, or air hunger. Due to the somatic underpinnings of PD, a major focus has been placed on interoceptive signaling and it is recognized that dysfunctional body-to-brain communication pathways promote the initiation and maintenance of PD symptomatology. While body-to-brain signaling can occur *via* several pathways, immune and humoral pathways play an important role in communicating bodily physiological state to the brain. Accumulating evidence suggests that neuroimmune mediators play a role in fear and panic-associated disorders, although this has not been systematically investigated. Currently, our understanding of the role of immune mechanisms in the etiology and maintenance of PD remains limited. In the current review, we attempt to summarize findings that support a role of immune dysregulation in PD symptomology. We compile evidence from human studies and panic-relevant rodent paradigms that indicate a role of systemic and brain immune signaling in the regulation of fear and panic-relevant behavior and physiology. Specifically, we discuss how immune signaling can contribute to maladaptive body-to-brain communication and conditioned fear that are relevant to spontaneous and conditioned symptoms of PD and identify putative avenues warranting future investigation.

## Introduction

A complex interplay and engagement of the central nervous system (CNS) and periphery is key to the genesis of emotional responses (1). Peripheral modulation of the-emotional responses was first postulated early under the peripheral feedback theory by William James (2) and later championed as interoceptive emotional modulation by several investigators (3, 4). Dysregulated interoceptive processing and maladaptive emotional responses are a hallmark of fear –associated disorders, particularly Panic Disorder (PD), a debilitating psychiatric illness which occurs in ∼4–8% of Americans (5–7). PD typically begins in the second decade of life (8) and is second only to major depressive disorder in terms of associated debility among psychiatric conditions in the United States (9). PD is characterized by recurrent panic attacks that consist of incapacitating periods of acute-onset respiratory, cardiovascular, gastrointestinal autonomic, and cognitive symptoms. According to the *DSM-5* (10), recurrent panic attacks in PD are categorized as being either spontaneous (unexpected) or cued (expected). Recurrence of panic attacks leads to anticipatory anxiety, conditioned fear and avoidance of panic contexts, cues and reminders leading to compromised functioning and disability (11–15). Current treatments have limited therapeutic efficacy and a delayed onset of action (15–17). While studies in the past few decades have improved our understanding of panic neurobiology [reviewed in: (18–20)], the mechanistic basis of spontaneous panic and sustained fear is still poorly understood yet could lead to improvements in treatment outcomes.

The association of PD with dysregulated interoceptive processing suggests an important role for body-to-brain signaling in PD pathology. While body-to-brain signaling can occur *via* several pathways, immune and humoral pathways play an important role in communicating bodily physiological state to the brain (21). Immune activation in conjunction with humoral interoceptive mechanisms can mediate discrete changes in brain and behavior that may predispose to psychiatric disorders (21–24). Strong evidence supports a role for the immune system and associated cellular mediators in regulation of behaviors associated with depression and anxiety such as sickness behavior, anhedonia and learned helplessness [reviewed in (22–25)]. Accumulating evidence suggests that neuroimmune regulatory mechanisms also play a role in fear and panic-associated disorders, although this has not been well studied. Interestingly, mounting epidemiological evidence supports a high comorbidity of PD with inflammatory conditions such as Crohn’s disease, asthma, inflammatory bowel syndrome (IBS) and fibromyalgia (26–31), suggesting a potential role of dysregulated immune signaling in PD pathology. However, our understanding of the role of immune mediators in the etiology and maintenance of PD remains limited.

Thus, in the current review, we attempt to summarize findings that support a role of immune dysregulation in PD symptomology. We compile evidence from human studies and panic-relevant rodent paradigms that indicate a role of systemic and brain immune signaling, in the regulation of fear and panic-relevant behavior and physiology. Specifically, we discuss how immune signaling can contribute to maladaptive body-to-brain communication and conditioned fear that are associated with spontaneous and conditioned aspects in PD onset and maintenance.

## Relevance of interoception and conditioned fear in panic disorder

**FIGURE 1:**
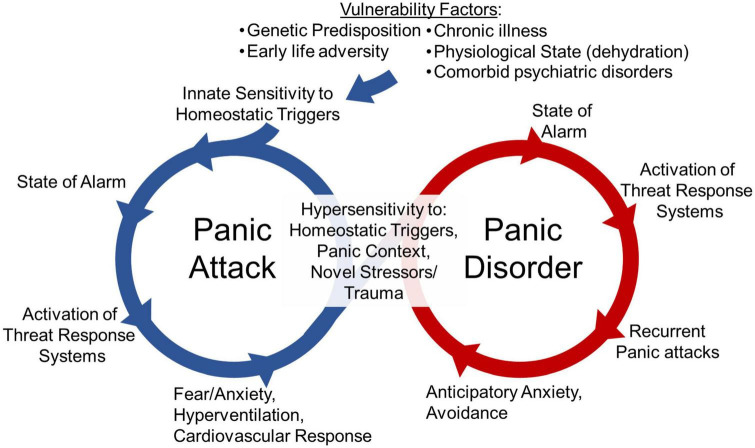
The cycle of panic disorder: Vulnerability factors such as genetic predisposition, early life adversity, chronic illness, physiological state (i.e., dehydration), or other psychiatric disorder diagnosis associate with innate sensitivity to homeostatic triggers. These homeostatic triggers are threats to internal homeostasis that lead to a heightened state of alarm, the activation of threat responses systems and a panic attack. The emotional, behavioral and physiological responses occurring during panic attacks such as fear, anxiety, hyperventilation and cardiovascular responses are evoked in an effort restore physiological homeostasis. The engagement of these systems and the conditioned responses to contexts where panic attacks occurred can lead to hypersensitivity to future homeostatic triggers, novel stressors or trauma, and exposure to previous panic contexts. This can lead to a cycle of recurrent panic attacks, and anticipatory anxiety and avoidance of panic-associated contexts, that ultimately facilitates the development of panic disorder.

Clinical observations and collective evidence from challenge studies in the laboratory, neuroimaging, symptomology, treatment responses and translational animal models have led to an increased understanding of PD (11, 12, 14, 18–20, 32–41). As illustrated in Figure 1, PD frequently originates with patients experiencing spontaneous panic attacks that seem to occur without an explicit trigger. Over time, PD develops as the result of associative conditioning processes that lead to fear and phobic avoidance as well as anticipatory anxiety of future attacks (15). Thus, to improve our understanding of PD and develop novel treatments, clinical and preclinical work has sought to understand the mechanisms underlying these various aspects of panic pathology, including both spontaneous panic attacks and attacks driven by conditioned responses, as well as the relationship between these processes.

Panic disorder is highly heterogenous with variable symptom profile and intensity in panic episodes experienced by the same individual and across patients (14, 15, 42). Interestingly, PD is unique among anxiety disorders in that fear and anxiety associated with panic are primarily directed toward somatic symptoms (32). In these individuals, body sensations or physiological signals linked to risk of suffocation (e.g., dyspnea, breathlessness, or air hunger) elicit dysfunctional defensive responding leading to anxious apprehension, fear, panic, contributing to the persistence of PD symptomatology. Due to the somatic underpinnings of PD, a major focus has been placed on interoceptive signaling and body-to-brain communication pathways, as interoceptive inputs serve an important regulatory function in generation of adaptive behaviors and physiology key to emotional regulation (3, 4, 37). Indeed, strong evidence now supports a primary role of homeostasis and interoception “an individual’s sensing and monitoring of the physiological condition of the body itself” in driving panic attacks (3, 4, 37, 43). In particular, it has been proposed that while the *expected* or *cued* panic attacks in PD are triggered by exteroceptive triggers (i.e., context of previous panic attack or other unrelated stressors, or traumatic experiences), the *unexpected* or *spontaneous* panic attacks may be provoked by interoceptive sensory triggers caused by fluctuations in the internal milieu that challenge homeostasis (37).

This theory of interoceptive signaling driving PD is primarily based on observations that patients with PD show heightened sensitivity to homeostatic disturbances, particularly those that induce acidosis such as CO_2_ inhalation and sodium lactate infusion (18, 38, 40, 44–62). In people with PD, these threats to internal homeostasis drive increased fear, respiratory, and cardiac responses that can result in panic attacks, suggesting this heightened sensitivity may confer vulnerability to panic attacks (38, 58). The ability to evoke panic attacks in clinical populations within the lab has greatly improved our understanding of the behavioral, emotional and physiological aspects of panic attacks. It has also allowed researchers to investigate the effects of pharmacological treatments or other therapies (i.e., cognitive behavioral therapy, etc.). Additionally, it has allowed for quantification of molecular biomarkers within blood or saliva immediately before and after panic attacks.

CO_2_ inhalation is the interoceptive stressor most commonly used to probe the mechanisms underlying panic attacks and PD. In this model, individuals are exposed to either low-dose CO_2_ inhalation (5–7.5% CO_2_) or a single beath of high-dose 35% CO_2_ by breathing in air composed of non-hypoxic levels of oxygen (∼21%) and varied nitrogen levels used to balance differing CO_2_ percentages (19, 34, 38, 45, 46, 49, 55, 56, 58, 59, 63, 64). CO_2_ inhalation evokes fear, anxiety and physiological responses in both patients with PD and in healthy individuals (38, 45, 46, 55). Responses to CO_2_ inhalation increase in intensity as the concentration of CO_2_ increases and tend to be stable across time (53). Interestingly, CO_2_ sensitivity seems to lie on a spectrum. Compared to individuals without PD, patients with PD show a left-shifted CO_2_ inhalation response curve, presenting with heightened emotional and physiological responses, and a greater likelihood of a resultant panic attack across all CO_2_ inhalation concentrations. First-degree family members of patients with PD present with an intermediate CO_2_ sensitivity phenotype between healthy individuals and those with PD suggesting there may be a genetic influence on this phenotype (38). Although not clearly defined, predisposition factors associated with PD such as genetics, early life adversity, underlying respiratory abnormalities and other factors are thought to promote sensitivity to interoceptive stressors like CO_2_ inhalation (18, 20, 52, 53, 61, 65, 66). Given the strength of the association between sensitivity to interoceptive stressors and PD, studies seeking to understand the mechanisms driving CO_2_ sensitivity may improve our understanding of PD and lead to identification of novel treatments.

Although spontaneous panic attacks are key to panic etiology, the development to PD also involves associative conditioning processes leading to fear and phobic avoidance (15, 32, 37, 67, 68). This can be particularly detrimental to quality of life in patients as they begin to avoid situations or contexts they believe may elicit panic attacks. Studies exploring conditioned fear responses, particularly contextual associations, have improved our understanding of PD and aided the development of behavioral therapies (32, 37, 67, 68). Studies investigating the transition from initial spontaneous panic attacks to development of conditioned fear are more limited yet understanding this transition may improve our ability to prevent the development of PD or improve treatments. Previous evidence suggests that initial panic attacks associated with dysfunctional interoceptive body-to-brain signaling could lead to sensitized fear-arousal-stress regulatory circuits to promote the chronicity and maintenance of PD (see Figure 1). However, the specific mechanisms by which dysfunctional interoceptive signaling could promote sensitized fear-arousal-stress responding are unclear.

Given the relevance of systemic and CNS interactions and crosstalk in panic genesis, the immune system provides an interesting regulatory pathway that can provide mechanistic insights on panic physiology. Historically, interoceptive signaling has largely focused on neural-mediated mechanisms (4, 33, 37, 41) however humoral signals are increasingly being recognized as important mediators in communicating bodily physiological state to the brain (21). Immune mediators and pathways can regulate neuronal activity and function leading to altered behavior and physiology (69–72). Reciprocally, sympathetic and neuroendocrine signals from the brain regulate immune response and function (24, 73–75). Relevance of immune mediators and inflammation in fear associated disorders such as PTSD is recognized (75–77). Whether dysfunctional immune signaling in the periphery or brain contributes to panic symptomology has not been systematically assessed. In the following sections we provide support for a potential PD-neuroimmune link based on evidence from genetic studies and cytokine measurements in human studies as well as panic-relevant animal paradigms simulating interoceptive and conditioned fear aspects of PD.

## Immune contributions in panic and fear: Clinical evidence

Mounting evidence from the clinic supports a potential role of dysregulated immune function in PD. Most work has focused on identifying specific genes, epigenetic associations or alterations in immune effectors comparing individuals with PD to those without a PD diagnosis that we will discuss below:

1) Immune-associated genes in Panic Disorder

Genetic studies indicate a heritability of PD at about 40% (78) suggesting a strong role for genetic variance in mediating risk to develop PD. Identifying the specific genes or gene clusters (groups of genes involved in similar functions or pathways) associated with PD could help improve our understanding of the mechanisms underlying PD and point to putative therapeutic targets. Multiple genetic studies have identified associations between immunomodulatory genes and PD. Single nucleotide polymorphisms (SNPs) within the INF-γ (+ 874 A/T), TNF-α (-308 G/A), and IL-10 (-1082 G/A) genes were investigated in patients with PD (79). The group reported that the G allele in IL-10-1082 G/A might have a protective role in reducing the manifestations of PD in female patients. IL-10 is an anti-inflammatory cytokine inhibiting the generation of several inflammatory cytokines (80, 81). Interestingly, IL-10 inhibits the nearly ubiquitous expression of indoleamine 2,3-dioxygenase (IDO), an enzyme responsible for directing tryptophan degradation, a pathway that has been implicated in anxiety disorders (82–84). Another study found an association of polymorphisms in the IKBKE (inhibitor of kappa light polypeptide gene enhancer in B cells, kinase epsilon) gene in patients with PD (85). IKBKE is involved in regulation of innate immunity, inhibiting NF-kappa B signaling in response to inflammatory cytokines, particularly IL-1 (85, 86), suggesting a potential role of innate immune signaling in PD. Association of a MASP-2 YA haplotype and Mannan-binding lectin (MBL) deficiency was reported in patients with PD, an observation explained to be associated with innate immune alterations that may increase susceptibility for infections and autoimmune states due to their roles in complement activation (87). In strong support of an immune dysfunction in PD, Shimada-Sugimoto et al. (88), performed pathway analyses in order to overcome the limitations of conventional single-marker analysis in identifying associated SNPs with modest effects. Using multiple pathway analyses the group reported that pathways related to immunity show the strongest association with PD. For further investigation, the group studied *HLA* polymorphisms in candidate susceptibility genes HLA-B and HLA-DRB1, associated with the major histocompatibility complex (MHC) and immune dysfunction in PD patients and control subjects (89). Patients with PD were significantly more likely to carry HLA-DRB1 further supporting links to immune regulation and genes involved in immune-related pathways are associated with PD.

In addition to gene polymorphism and pathway analysis studies, epigenetic contributions have been investigated in the context of immune modulation. A recent study investigated whether aberrant DNA methylation of inflammation-related genes was associated in the development of PD (90). Methylation levels of CCL3, CRP, CSF2, CXCL8, IFNG, IL12B, IL1A, IL-4, IL-6, TNF was investigated. Significantly higher methylation levels of the IL-4 gene were observed in PD patients than control subjects. Importantly, the methylation levels of IL-4 gene showed a significant positive correlation with the severity of panic and anxiety (90). In another study, (91), a significant association between panic severity score and methylation levels of Asb1, a member of the suppressors of cytokine signaling (SOCS) family was observed in PD subjects, suggesting a role of epigenetic factors. Furthermore, in a follow up mouse study, the authors found a correlation between peripheral Asb1 and IL-1β mRNA expression after acute social defeat stress suggesting a relationship between Asb1, IL-1β and stress responding (91). Assessment of T cell receptor excision circles (TRECs), Forkhead-Box-Protein P3 gene (FOXP3) methylation of regulatory T cells (Tregs) and relative telomere lengths (RTLs) was conducted in patients with PD and age- and sex-matched healthy controls in order to test for a potential dysfunction and premature aging of the immune system (92). Significantly reduced TRECs in PD patients and FOXP3 hypermethylation in female patients with PD was observed, reflecting immune system-related deficits in PD.

Other studies support differences in the expression of immune-associated genes in panic pathology. Our lab found significantly higher expression of immunomodulatory gene T-cell death associated gene 8 (TDAG8) in peripheral monocytes collected from PD subjects and healthy volunteers (93). TDAG8, also expressed in brain microglia, is an acid sensing GPCR regulating CO_2_-associated fear (94). Interestingly, a significant positive correlation was observed between monocytic TDAG8 expression and panic symptom severity score and TDAG8 expression was lower in individuals with symptom remission, suggesting potential utility of TDAG8 as a treatment response biomarker (93). Lastly, one group, Maron et al. (95) conducted gene expression profiling following cholecystokinin CCK-4, a commonly used panic provocation agent challenge in control subjects. Interestingly, several immune regulatory genes showed alterations between “panickers” and “non-panickers,” suggesting that acute panicogenesis may engage immune system targets.

Collectively, genetic evidence suggests dysregulation of immune function and possibly epigenetic mechanisms in panic etiology. Although some association with immunomodulatory genes have been reported in PD, more follow up investigation is warranted.

2) Immune effectors in PD

Measurement of alterations in immune mediators such as cytokines, chemokines and T cells have been conducted in individuals with PD. Several studies have reported altered cytokine concentrations in PD, however directionality and targets assessed differ between studies and are not always consistent. In general, a broad spectrum of cytokines appear to be upregulated in PD (96, 97) suggesting dysregulated immune signaling, however, specific associations and contribution to panic physiology is not well understood. Due to variability in study layouts and for correct interpretation, we have divided available evidence into measurements performed at baseline (no challenge) or following a panic-relevant challenge or stressor.

i) Immune alterations in panic disorder under non-challenge conditions

Proinflammatory mediators within the interleukin (IL) family have been the most studied in the context of PD with measurements reported on IL-6, IL-1β, IL-2, IL-3, IL-12, IL-10, and TNFα [see (97)]. Multiple studies have shown elevated IL-6 concentrations in the serum of PD subjects (96, 98–100). One study showed that while patients with both PD and generalized anxiety disorder (GAD) showed higher IL-6 compared to healthy individuals, PD subjects had even greater IL-6 than individuals with GAD (98). Interestingly, the utility of IL-6 as a potential treatment response biomarker is supported by significantly lower IL-6 concentrations in individual with remitting symptoms compared to those with current panic symptoms (99). Furthermore, another study observed as association of pretreatment IL-6 with poor treatment response (100). However, other studies have reported no significant changes in IL-6 in PD patients (101, 102), suggesting that factors other than a panic diagnosis may contribute to IL-6 alterations.

The IL-1 family of cytokines have also been investigated in PD. A study by Brambilla et al. (103) measured IL-1β plasma concentrations before and after treatment with alprazolam, and reported significantly higher IL1β both before and after treatment (103). However, other studies have reported no associations of IL-1α/IL-1β with PD (102, 104). These inconsistencies have been contributed to differences in assay methodologies (97).

C-reactive protein (CRP), an inflammatory marker shows significant elevation in patients with PD as compared to healthy controls (105). Following 8 weeks of selective serotonin reuptake inhibitor (SSRI) treatment, CRP concentrations decreased only in treatment-responsive individuals. Additionally, the authors found reduced γ globulin and higher cortisol levels in PD patients compared to controls prior to initiation of treatment. Collectively, these observations suggest engagement of the acute phase response in PD.

Measurement of broad spectrum panel of cytokines and inflammatory markers such IL−6, IL−1α, IL−1β, IL−8, MCP−1, MIP−1α, Eotaxin, GM−CSF, and IFN−α revealed that 87% of individuals with PD or PTSD had six or more detectable levels of these cytokines, compared with only 25% of control (96). In PD patients compared to controls, 17 of 20 cytokines and chemokines examined showed significant elevation, suggesting that PD may associate with a generalized inflammatory state and that specific “inflammatory signatures” may differ on an individual basis. This could associate with the general heterogeneity in symptomology shown within PD patients.

Among other members of the interleukin family, higher IL-2 concentrations were reported in PD subjects compared to healthy controls by one study (104), however no associations were reported by other studies (101, 102, 106). One study reported increased IL-18 concentrations in PD the magnitude of which was comparable to a concurrently tested group of depression subjects (107).

ii) Immune alterations post stressor in patients with PD

The ability to respond appropriately to stressors and adapt is critical to survival by increasing alertness and preparing the body to fight (108). Engagement of stress responses with the immune system may have originated from a need to prepare the body to fight infection from wounds (109). Mounting evidence suggests that immune system engagement may also regulate fear learning which may be beneficial to survival by helping an individual remember that a fearful environment should be avoided (109). Immune responses to stressors occur in healthy individuals, but evidence suggests these responses may be dysregulated in those with psychiatric diagnoses (22, 70, 73, 110). As both homeostatic and psychogenic stressors are relevant to panic physiology (Figure 1), investigating immune-associated alterations following panic-relevant stressors in individuals with PD can provide valuable information.

To date, only one study directly studied alterations in immune factors pre- and post -CO_2_ inhalation (101), a well-established interoceptive stressor in PD described above. No significant differences in either baseline or post CO_2_ concentrations of TNF-α, IL-6, IL-8, IL-10, IL-1RA, soluble sIL-2R, soluble sIL-6R or positive acute phase protein haptoglobin were observed. Lipopolysaccharide-stimulated cytokine production (TNF-α, IL-6, IL-1β, IL-10, IFN-γ) in whole blood was comparable for PD patients and their matched controls. Limitations of the study suggested by the authors included: the low number of subjects and a gender-biased sample with underrepresentation of women. Given the relevance of panic provocation challenges in PD, more larger “n” studies are warranted; not only for CO_2_ inhalation, but other interoceptive challenges such as sodium lactate.

Dysfunctional stress response systems such as the hypothalamic pituitary adrenal (HPA) axis and sympathetic nervous system (SNS) have been reported in PD [reviewed in (111)]. These stress response systems have bidirectional regulatory associations with the immune system, with either system engaging the other to regulate immune and stress responses (24, 112–114). Accumulating evidence indicates stress-neuroimmune interactions in anxiety disorders including PD (115). Given strong evidence of immune dysregulation within PD patients under non-stressful conditions, it is important to determine whether immune responses to stressful stimuli are dysregulated and whether immune dysregulation could contribute to CO_2_ sensitivity in patients with PD.

Exposure to acute psychological stressors have previously been reported to show engagement with the immune system, particularly cytokine release. For example, public speaking or performing complex tasks in public elevated plasma cytokines and CRP (116–118). One study compared immune mediators in healthy individuals and those with PD following the Trier Social Test (TSST) (119). They found PD patients have higher baseline and post stressor concentrations of IL-10, and also show blunted cortisol responses. Interestingly, peak IL-6 concentration associated with PD symptom severity. Heightened startle responses have also been reported in PD patients compared to healthy controls only under fear-associated threat conditions (120). Interestingly, there is an association between heightened startle reactivity and inflammation (75).

Collectively, evidence suggests altered immune mediators in PD, that may represent underlying immune dysfunction, however, additional studies especially following panic-relevant triggers and stress challenges are required for understanding their association with PD physiology. Human studies are limited and cannot provide mechanistic information on brain neuroimmune alterations and association with panic-relevant behaviors. Furthermore, it is difficult to probe neurocircuits orchestrating body to brain signaling. In the following section, we discuss selected paradigms that may be relevant to understanding the mechanistic association of immune dysregulation and panic associated behavior and physiology.

## Understanding panic-fear-neuroimmune links: Relevant translational models

Several panic-relevant rodent models have been developed over the last few decades that provide valuable insights on panic pathophysiology. Consistent with the importance of uncued/spontaneous and cued triggers in generating behavioral and physiological responses in PD, these models have focused on homeostatic and stress challenges, respectively [reviewed in (18–20, 34, 48, 52, 121–124)]. Previously reviewed literature has primarily focused on acid-base homeostasis, PD-relevant neurotransmitter systems, region-targeted interventions, genetic and transgenic manipulations. Despite emerging evidence from human studies, contribution of immune mechanisms in panic and fear has not been systematically investigated using translational paradigms. In this section, we assess selected rodent paradigms that provide information on the potential role of immune cells, targets and signaling that may regulate panic-relevant responses and provide mechanistic insights on panic-fear-immune links (see Table 1).

**TABLE 1:** Animal studies of immune regulation of spontaneous/conditioned fear.

Model	PD-relevant phenotype and effect	Potential immune effectors	Brain region(s) implicated	References
**Panic-relevant (spontaneous)**				
CO_2_ Inhalation	Freezing, respiration, blood pressure	TDAG8, IL-1β, IL-1RA	SFO	Vollmer et al. (94)
	Freezing, rearing	IL-1β, IL-1R1, IL-1RA	SFO, mPFC, amygdala, PAG	McMurray et al. (152)
		Microglia	Nucleus tractus solitarius, locus coeruleus	Marques et al. (169)
**Panic-relevant (conditioned fear)**				
Fear conditioning (FC) (without prior stress; no follow up behavior)	Conditioned fear (freezing), extinction (freezing)	IL-6		Young et al. (76)
	Contextual extinction acquisition (freezing)	TNF-α	Whole brain and hippocampus	Yu et al. (203)
	Auditory extinction acquisition (freezing)	IFN- α, IL-1β, TNF-α	Amygdala	Bi et al. (215)
	Extinction acquisition (freezing)	IL-6	Amygdala	Hao et al. (202)
	Contextual fear extinction (Freezing); Open field (center duration), Elevated Plus Maze (open arm time)	Nlrp3 inflammasome: cleaved Casp1, IL-1β, and TNFα	Hippocampus	Dong et al. (204)
**Models of panic vulnerability (stressors exacerbating later panic-relevant outcomes)**				
Early life stressor: neonatal maternal separation		Impaired response to influenza infection: IL-1, IL-6, IL-12, IFN-g, and TNFa mRNA in lung (IL-1, TNFa and IFNg only in females) corticosterone		Avitsur et al. (228)
	Open field (center time), forced swim test (immobility)	CD8^+^ T cells, spleen T cell CD4/CD8 ratio, thymocytes, corticosterone		Roque et al. (229)
		CD8/CD4 cell ratio, natural killer cells, lymphocytes (monkeys)		Lubach Coe and Ershler (230)
	Cardio/respiratory control	Synaptic pruning *via* microglia	Medulla, Nucleus tractus solitarius, dorsal motor nucleus of vagus	Baldy et al. (168)
	CO_2_ sensitivity: respiratory response to 6% CO_2_ inhalation	N/A		Luchetti et al. (164), Cittaro et al. (163), Giannese et al. (166), Battaglia et al. (126), D’Amato et al. (65), Giannese et al. (166)
Early life stressor: repeated cross fostering	CO_2_ sensitivity: respiratory response to 5% CO_2_ inhalation	N/A		Genest et al. (167)
		Neonatal dexamethasone treatment increases susceptibility to experimental autoimmune encephalomyelitis in adult rats corticosterone, TNFα, IL-1β release Changes peripheral T cell Vbeta repertoire	Peritoneal macrophages	Bakker et al. (231), Bakker et al. (232)
		changes in microbiota		Daft et al. (233)
	Anxiety-relevant behaviors in males			Bartolomucci et al. (234)
	Fear conditioning (freezing)	IL-1β (Astrocytes)	Hippocampus	Jones et al. (208), Jones et al. (210), Jones et al. (210)
Stress-enhanced fear learning (SEFL)	N/A	IL-1β, IL-18, IL-6, IL-10, monocyte chemotactic protein (MCP-1), DAMPs (uric acid and Hsp72)	Plasma	Maslanik et al. (235)
Inescapable shocks	Fear memory	peripheral T lymphocytes		Clark et al. (205)
Predator stress	Fear conditioning acquisition (freezing), 2-way Avoidance/Escape (active responding, escape failures), motor activity, fatigue	plasma TNF; corticosterone; dysregulated immune pathway gene expression	PFC, Amygdala, Periphery (plasma/spleen)	Azzinnari et al. (236)
Social defeat/chronic social stress	Anxiety-relevant behavior (Open field test, elevated plus maze)	Monocytes, macrophages, IL-1β	Spleen, whole brain	Lisboa et al. (182)
	Tachycardia, heart rate variability			Morais-Silva et al. (192)
	Anxiety-relevant behavior (open field test, light-dark preference)	IL-1β, IL-1R1, TNFα, IL-6	Brain	Wohleb et al. (184)
Social defeat/chronic social stress restraint stress	Social exploration	IL-1R1	Hippocampus	DiSabato et al. (185)
Inescapable shocks	Anxiety-relevant (Open field test)	IL-1β, monocytes		McKim et al. (186)
Predator stress	Anxiety-relevant (light-dark preference)	Macrophages, IL-1b, IL-6, TNFa	Brain	Wohleb et al. (187)
Social defeat/chronic social stress		Monocyte-derived microRNA106b∼25		Pfau et al. (188)
		IL-6	periphery	Hodes et al. (189)
	Social interaction	Dihydrocaffeic acid (DHCA) and malvidin-3′-O-glucoside (Mal-gluc), IL-6		Wang et al. (190)
		IL-6	Nucleus accumbens	Menard et al. (191)
	Bradypnea		Dorsal medial hypothalamus, nucleus of the solitary tract	Brouillard et al. (194)

1) CO_2_ inhalation and microglial acid sensing by TDAG8

As described in preceding sections, homeostatic triggers that promote acid-base imbalance such as low-dose, non-hypoxic CO_2_ inhalation induce intense fear, anxiety, physiological responses and panic attacks in PD subjects (18, 19, 34, 38, 41, 47, 56). Several investigators including our laboratory, have used CO_2_ inhalation to simulate panic-relevant behavior and physiology in mice and rats (48, 65, 94, 125–133). The partial pressure of CO_2_ in the blood and CNS increases following CO_2_ inhalation challenge. In the extracellular fluid, CO_2_ is hydrolyzed to carbonic acid (H_2_CO_3_) by carbonic anhydrase which readily dissociates into bicarbonate (HCO_3_^–^) and H^+^ (134). The resulting acidosis constitutes a homeostatic threat that is sensed by acid-sensing chemosensors in the brain and periphery (18, 19, 34, 38, 41, 47, 56). This model has strong translational value due to its strong face, predictive and etiological validity [discussed in: (18, 19, 48, 127)].

Given its strong translational value, CO_2_ inhalation in rodents is commonly used to investigate mechanisms underlying PD to simulate panic-relevant behaviors and physiology. To date there have been somewhat limited investigations into the role of immune dysregulation in mediating PD-relevant behaviors and physiological outcomes in CO_2_ inhalation models. However, support for immune dysregulation in these models is growing. Our lab cloned acid-sensing G protein coupled receptor, T- cell death associated gene-8 (TDAG8) in rodent brain (135), and reported TDAG8 expression on microglia, innate immune cells of the CNS (94), that are recruited in physiological responses to homeostatic fluctuations (136–140). On sensing subtle imbalance in ionic homeostasis, microglia transform rapidly from a resting to an activated state in accordance with their role in maintenance of the CNS microenvironment. Interestingly, TDAG8-expressing microglia were enriched within the sensory circumventricular organs (CVOs) (94). Sensory CVOs such as the subfornical organ (SFO) are integrative sites that lack a blood-brain barrier and have access to systemic and CNS compartments for maintenance of homeostasis (141). Importantly, the SFO has been identified as a site where interoceptive stimuli can be sensed and relayed to panic-generating CNS areas, and has been implicated in regulating panic-relevant responses to other panicogens such as intravenous lactate (142–144). More directly, our data showed targeted infusion of acidified artificial cerebrospinal fluid (aCSF; a homeostatic stress) into the SFO triggers fear-relevant behaviors like freezing; an effect which is dependent on TDAG8 (145). Our data further revealed the necessity of microglial acid-sensor TDAG8 to orchestrate CO_2_-evoked behavioral (freezing) and cardiovascular responses relevant to panic *via* microglial activation, IL-1β signaling, and SFO neuronal firing (94). Thus, the SFO likely represents a primary locus for detecting interoceptive triggers in “body-to brain” transmission of panic.

Recruitment of SFO immunomodulatory mechanisms in panic-relevant outcomes is novel, although not surprising, as the SFO is also known to drive immune responses and sickness behaviors, particularly fever (141, 146–148). The SFO also regulates blood pressure (149) as well as respiration (150), and previous studies reported IL-1β-IL-1R signaling mediated cardiovascular activation *via* the SFO (151). More recent observations from our lab show significant attenuation of CO_2_-evoked freezing following SFO-targeted infusion of the IL-1 receptor antagonist, IL-1RA (152), suggesting a primary role of SFO IL-1R1 signaling in CO_2_-associated fear behaviors. Notably, the IL-1R1 receptor is localized on endothelial cells within the SFO (152, 153) and is required for CO_2_-evoked activation of SFO neurons (94). The SFO could be mediating these effects though its direct projections to brain areas mediating defensive behaviors and physiological responses relevant to panic such as the hypothalamus, prefrontal cortex, BNST, and periaqueductal gray (154, 155). Collectively, our data highlight a contributory role of neuroimmune signaling and specialized sites such as the SFO in regulation of panic-relevant behavior and physiology.

2) Early Life Stress- CO_2_ respiratory dysfunction paradigm

Strong evidence supports significant variance and individual differences in responding to CO_2_ inhalation (38, 47, 156). People with panic lie at one extreme of the sensitivity distribution, showing paroxysmal hyperventilation and panic when they undergo CO_2_ challenges (38, 52, 58, 61, 157). Modeling CO_2_ sensitivity in rodents as a proxy for human panic has unique strategic advantages such as an opportunity to dissect mechanistic contributions. Studies performed by Battaglia and coworkers have highlighted gene x environment interactions in mediating increased CO_2_ sensitivity [reviewed in (158–160)]. Chronic stress in the form of early life adversity for example childhood parental loss, contributes to these relationships (158, 161), and stressful life events occurring in childhood-adolescence heighten young adults’ CO_2_ sensitivity (162).

Translationally relevant paradigms such as neonatal maternal separation (NMS) and repeated cross fostering (RCF) model have effectively simulated the effects of early life stress on CO_2_ sensitivity and provided mechanistic insights [reviewed in (158, 160)]. Heightened ventilatory response to CO_2_ inhalation is observed in adult animals with a history of NMS or RCF as neonates (65, 126, 163–167), that may be regulated by gene x environment factors and epigenetic alterations in gene methylation patterns (163, 166). Interestingly, significant increase in microglial density and reduced arborization was observed within the nucleus of the solitary tract (cNTS) and the dorsal motor nucleus of the vagus (DMNV), two key areas regulating breathing (168) suggesting that NMS may compromise microglial ability to perform optimal synaptic pruning that could lead to aberrant respiratory control. In a more recent study (169), the group reported a significant effect of ovarian hormones on microglial activation within in CO_2_/H^+^ sensing brain stem areas, suggesting that hormonal fluctuations may influence anomalies of respiratory control *via* neuroimmune mechanisms. Given that effects of early life stress on immune signaling, neuroimmune physiology and function are well established [for review see (110, 170–172)], more investigation is warranted on the delineating the links between ELS-immune signaling and panic-relevant physiology.

3) Chronic Social Defeat paradigms - body to brain immune signaling in anxiety and fear

As described in preceding sections, exposure to exteroceptive stressors and aversive contexts can facilitate the recurrence of panic attacks. Furthermore, dysregulation of stress response systems has been implicated in PD, particularly the sympathetic nervous system that regulates the immune system *via* innervation of the spleen and lymphoid organs [Webster Marketon and Glaser (173); Dantzer (174)]. A primary role of chronic stress mediated immune dysfunction and CNS remodeling has been recognized in the genesis of psychiatric illnesses, including mood and anxiety disorders [reviewed in (24, 71, 72, 175)]. Recurrent or chronic stress engages intrinsic neuronal pathways that lead to physiological effects, such as neuroendocrine and sympathetic activation (176, 177). These systemic responses to stress further promote the release and trafficking of myeloid cells with enhanced inflammatory potential into various organs, including the brain (178, 179). Preclinical rodent models of stress-induced behavior and physiology can provide important information on brain-to-body as well as body-to-brain communication mechanisms. In this regard, psychosocial stress paradigms, particularly repeated social defeat (RSD) stress exposure [reviewed in (114, 179–181)] have yielded important mechanistic information on body-brain neuroimmune crosstalk in regulating fear extinction (182), anxiety and social avoidance behaviors [reviewed in (179)] that are relevant to persistent fear, anxiety and comorbid depression observed in PD. Coordinated events including activation of brain threat appraisal sites, microglial activation and peripheral immune signaling is reported to orchestrate RSD-mediated behavioral deficits. Exposure to RSD leads to increased sympathetic activation promoting elevated noradrenergic signaling in the periphery and increased production and release of glucocorticoid-insensitive monocytes into the circulation from the bone marrow and spleen [reviewed in (114, 183)]. Pharmacological and genetic intervention strategies using adrenergic receptor antagonists, microglial activation blockers, cell specific IL-1R transgenic mice highlight a key role of sympathetic activation, microglia-mediated trafficking of reactive IL-1β- releasing monocytes to the brain, and the recruitment of endothelial and neuronal IL-1R1 in promoting RDS induced expression of anxiety-associated behaviors and reduced social interaction (184–187). Although IL-1-mediated mechanisms appear to be important in the RDS model, it would be important to note that other cytokine mediators, like interleukin-6 have also been implicated in social defeat stress- induced blood brain barrier alterations and impaired social interaction behavior (188–191). In addition to adverse behavioral effects, social defeat exposure has long term effects on cardiovascular responses (192, 193) and aberrant respiratory patterns (194) that are of relevance to PD. Immune dysfunction and inflammatory mechanisms contribute to increased cardiovascular and autonomic activation in stressed mice (195, 196).

In summary, previous work has highlighted the relevance of social defeat stress paradigms to anxiety and depression physiology. However, the observed crosstalk of sympathetic activation, peripheral immune signaling and brain networks regulating behavior and physiological responses in this model may also relate to panic physiology.

4) Fear Conditioning paradigms

Pavlovian fear conditioning has been central to understanding the etiology of anxiety disorders such as PD [reviewed in (197)]. The clinical relevance of fear conditioning to PD is based on conditioned fear responses to panic-evoking stimuli and contexts, as well as generalized fear to resembling cues. Conditioning processes contribute by conferring fear and anxiogenic valence to these conditioning triggers that enable the maintenance of the disorder well after the termination of the unconditioned stimulus (i.e., the panic attack). The measurement of Pavlovian fear conditioning and extinction in rodents offers a relatively simple paradigm that is translatable as an approach to study biological underpinnings of fear-related disorders (198–201). While most rodent studies highlight the applicability of their findings to PTSD, they are also pertinent to PD symptomology. In this section we discuss evidence supporting a role of several immune factors and neuroimmune signaling in regulating conditioned fear associated behaviors. We discuss selected fear conditioning studies in this section excluding models of neurodegeneration, addiction or severe inflammatory insults.

A role of IL-6 in the maintenance of fear memory was reported using auditory cue fear conditioning, an effects dependent on fear extinction, suggesting that IL-6 and other IL-6 related pro-inflammatory cytokines may contribute to the persistence of fear memory (76). Another study reported impairment of acquisition and extinction of fear following intra-amygdala injection of IL-6 (202). Fear regulatory effects of other cytokines and T cells have also been investigated. Levels of TNF-α were increased in microglia from mice representing retention of fear memory, and returned to basal levels in mice representing extinction (203), suggesting that sustained fear is facilitated by microglial TNF-α signaling. A role of NLRP3 inflammasome activation and IL-1β has also been reported in the regulation of fear memory (204). Genetic knockout and pharmaceutical inhibition of the NLRP3 inflammasome enhanced the extinction of contextual fear memory. IL-1β administration inhibited extinction, while, IL-1RA (IL-1R antagonist) facilitated extinction. In addition to cytokines associated with innate immune signaling, the role of CD4(+) T cells in promoting fear responses by enhancing learning and memory processes has been reported (205). Lymphocyte deficient Rag2(-/-) mice showed attenuated fear responses in a cued fear conditioning paradigm compared to wild-type mice and reconstitution with CD4(+) T cells promoted fear learning and memory.

Stress-enhanced fear learning (SEFL), which encompasses both trauma and memory components in rodents, has emerged as a valuable preclinical model for PTSD [reviewed in (206)], and may be pertinent to the conditioned aspects of PD physiology. SEFL simulates the impact of traumatic stress in the form of several footshocks on subsequent fear conditioning to a single shock delivered in a novel context (207). Maladaptive behavioral outcomes following traumatic footshocks in the SEFL paradigm have been associated with immune dysregulation, specifically, a central role of hippocampal IL-1β has been reported (208–210). Exposure to footshocks induced a time-dependent increase in IL-1β expression within the hippocampal dentate gyrus, and IL-1RA treatment prevented the development of SEFL (208). Interestingly, hippocampal astrocytes were reported to be the source of IL-1β (209). To confirm a key role of astrocyte signaling in SEFL, the same group investigated the effects of glial-expressing DREADD construct [AAV8-GFAP-hM4Di(Gi)-mCherry] delivered in the dorsal hippocampus (210). Inhibition of astroglial G_*i*_ activation was sufficient to attenuate SEFL, suggesting that IL-1β signaling involves glial-neuronal interactions in stress potentiation of fear.

Collective evidence from these studies suggests that immune signaling has regulatory effects on fear learning and memory processes and that dysregulated immune status in PD could promote sustained and generalized fear.

## Potential immune links in panic disorder: Takeaways and future directions

**FIGURE 2:**
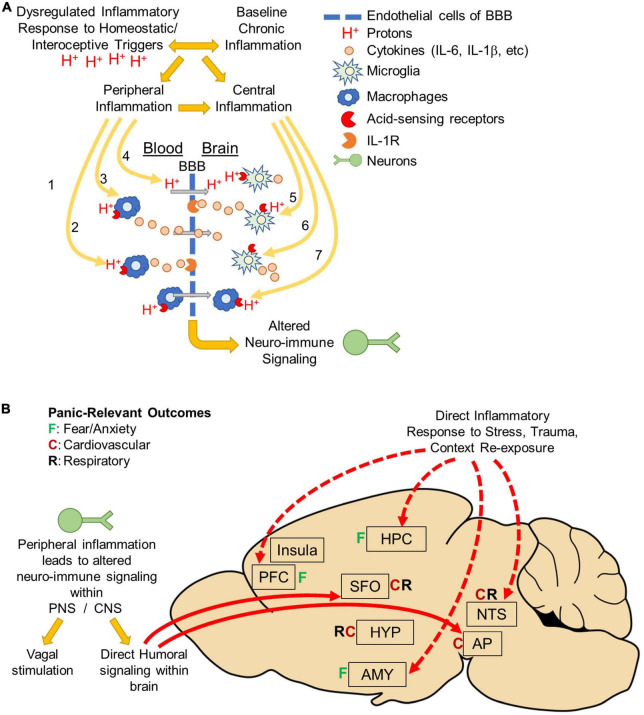
Body to brain signaling: Mechanisms of acidosis evoked peripheral and central inflammation: **(A)** panic disorder is associated with dysregulated inflammatory responses to homeostatic/interoceptive triggers like acidosis. Panic disorder is also associated with baseline chronic inflammation. Regardless of which inflammatory response occurs initially, either can facilitate the development of the other and result in peripheral and central inflammation. Acidosis could lead to altered neuroimmune signaling through a variety of mechanisms. In the periphery, acidosis may activate acid-sensing receptors on peripheral macrophages. This could lead to either (1) macrophage infiltration across the blood brain barrier (BBB), (2) cytokine release that activates cytokine receptors on endothelial cells, or (3) release of cytokines that cross the BBB. Peripheral acidosis could acidify the brain *via* protons (H^+^) that cross the BBB and activate acid-sensing receptors on microglia. Alternatively, acidosis could occur directly within the brain (5) activating acid-sensing receptors on microglia resulting in cytokine release and activation of cytokine receptors on endothelial cells or (6) directly on neurons or other cell types. It is also possible that acidosis could activate acid-sensing receptors on peripheral macrophages that have previously infiltrated the brain, possibly as a result of prior trauma or other risk factor (disease, etc.). Ultimately, Inflammation within the brain is thought to drive emotional, behavioral and physiological responses that occur during panic attacks and increase vulnerability to develop panic disorder. **(B)** Peripheral inflammation can alter neuronal activity either by neuro-immune signaling occurring either in the periphery or directly in brain. In the periphery, neuro-immune signaling could result in vagal stimulation that terminates in the nucleus tractus solitarius (NTS) and mediates cardiovascular and respiratory responses. There could also be direct humoral signaling within circumventricular organs the subfornical organ (SFO) or Area Postrema (AP). These areas are known to project directly to fear, respiratory and cardiovascular regulatory areas throughout the brain. Alternatively, inflammation could occur directly in the brain in response to stress (interoceptive/homeostatic or exteroceptive), trauma, or panic context re-exposure. Many studies have shown increased inflammation in fear-associated regions prefrontal cortex (PFC), hippocampus (HPC), amygdala (AMY) or the NTS. Inflammation in these areas could affect neuronal activity and drive the emotional, behavioral and physiological responses associated with panic disorder pathology.

Mounting evidence supports a role for neuroinflammation in PD, however, questions remain about the specific mechanisms and effectors contributing to pathology. Clinical observations suggest that PD associates with heightened peripheral inflammation at baseline. Patients with PD also present with hyperactive homeostatic stress responsivity, particularly in response to acid-base imbalance, which associates with dysregulated immune responses to stress. Our working model (see Figure 2) proposes a complex interplay between peripheral and central inflammatory mechanisms, and a hyperactive response system to homeostatic stress. While it is currently unclear if hyperactive homeostatic stress responsivity leads to dysregulated inflammatory signaling or if a heightened inflammatory state increases sensitivity to homeostatic stressors, it is likely the relationship is bidirectional with either facilitating the other. The exacerbation of sensitivity to homeostatic stressors like acidosis and peripheral inflammation then promotes the behavioral, emotional and physiological symptoms of panic attacks through neuroinflammatory mechanisms and dysregulated neuroimmune signaling within the brain and ultimately, leads to the development of PD.

An important question going forward involves the relationship between peripheral and central inflammation, and how inflammation reaches the brain to ultimately drive panic symptomology. Though it is unclear how inflammation could reach the brain in PD, homeostatic stressors like acidosis could lead to altered neuroimmune signaling within the brain through a variety of mechanisms (Figure 2A). In the periphery, acidosis may activate acid-sensing receptors on peripheral macrophages and lead to release of cytokines that either activate cytokine receptors on endothelial cells or cross the BBB to activate receptors directly within the brain. Alternatively, peripheral acidosis could acidify the brain *via* protons (H^+^) that cross the BBB and activate acid-sensing receptors on microglia. Another possibility is that acidosis occurs directly within the brain activating acid-sensing receptors on microglia resulting in cytokine release and activation of cytokine receptors on endothelial cells or directly on neurons or other cell types. The BBB generally protects against infiltration from peripheral immune cells, however mounting evidence suggests mood, anxiety and stress-associated psychiatric disorders like PTSD and depression associate with changes in BBB permeability (191, 211–213). In PD, it is possible that prior trauma, chronic stress or comorbidities such as PTSD or depression which associate with changes in BBB permeability could cause peripheral macrophages to penetrate the brain, which could then be activated by subsequent acidosis. It is also possible that repeated activation of peripheral macrophages could alter the BBB and lead to changes in how peripheral macrophages interact or engage with the peripheral-endothelial-BBB interphase. Future studies are needed to identify which, if any, of these mechanisms contribute to neuroinflammation in PD and determine whether targeting these mechanisms could improve treatment outcomes.

Future studies are also needed to identify how inflammatory signaling is translated into neuronal signaling within the brain to ultimately regulate panic symptomology. The conversion of immune signaling into neuronal signaling could originate either in the periphery or directly in brain (Figure 2B). In the periphery, neuro-immune signaling could result in vagal stimulation that terminates in the nucleus tractus solitarius (NTS) and mediates cardiovascular and respiratory responses (214). There could also be direct humoral signaling within circumventricular organs the subfornical organ (SFO) or area postrema (AP). These areas are known to project directly to fear, respiratory and cardiovascular regulatory areas throughout the brain (147). Alternatively, inflammation could occur directly in the brain in response to stress (interoceptive/homeostatic or exteroceptive), trauma, or panic context re-exposure. Many studies have shown increased inflammation in fear-associated regions prefrontal cortex (PFC), hippocampus (HPC), amygdala (AMY) or the NTS (75, 83, 204, 208, 209, 215–217). Inflammation in these areas could affect neuronal activity and drive the emotional, behavioral and physiological responses associated with PD pathology.

Another consideration is the level of inflammation associated with pathology. Historically, most studies on the role of innate immune cells within the brain have focused on the effects of high levels of neuroinflammation more typically associated sickness that can have more dramatic effects such as increased phagocytosis. Yet even mild perturbations in the immune milieu can have neuromodulatory effects. Innate immune cells contribute to homeostatic maintenance of the CNS and dysregulation of this functionality may lead to pathology (140). For example, microglia have an important role in the synaptic pruning (72). Dysregulated synaptic pruning could have lasting effects on neurotransmission and mounting evidence suggests an association of dysregulated synaptic pruning with psychiatric disorders like depression or autism. There is also an association between inflammation and increased oxidative stress in psychopathology which could exacerbate cell damage, alter cell signaling within the brain and result in behavioral changes (218–221). Additionally, at lower concentrations cytokines may act as neuromodulators within the brain, changing excitability and having more mild effects on cognition/memory/decision making rather than inducing robust sickness behaviors at higher concentrations (70). In the case of PD, a lack of evidence for drastic effects on immune function might be due to relatively mild changes on the innate immune system, resulting in changes to homeostatic maintenance or monocyte trafficking to endothelial cells at the BBB rather than increased cell death or infiltration of peripheral monocytes/T cells as a result of BBB degradation.

Lastly, the high level of co-morbidity between PD and inflammatory diseases such as multiple sclerosis (222), lupus (89, 223), asthma (30, 224), and irritable bowel syndrome (225), as well as other psychiatric disorders associated with inflammation like depression (6, 14, 226), PTSD (6, 227) and substance use disorders (6, 14) supports a role for dysregulated immune signaling in PD risk and pathology. However, the mechanisms driving high comorbidity are unclear and particularly complex as either PD or the inflammatory disorder may pre-date the other. For example, initial presentation with an inflammation-associated disorder could drive changes in homeostatic sensitivity and lead to increased risk for developing panic, innate sensitivity to homeostatic stressors could lead to development of a heightened inflammatory state and increase risk for developing inflammation-associated co-morbidities, or the same underlying inflammatory pathways may drive pathology in PD and commonly co-morbid inflammatory disorders and be dysregulated in affected individuals. Future studies are needed to determine the relationship between these disorders and the mechanisms through which one may lead to the other.

Overall, strong evidence supports the hypothesis that dysregulated body to brain signaling is central to the development of PD. Emerging clinical and preclinical evidence points to a strong role for dysregulated immune signaling in driving the dysfunctional body to brain signaling underlying spontaneous and cued panic attacks. However, further studies are needed to better understand neuroinflammatory mechanisms in PD.

## Author contributions

KM and RS conceived of the project, wrote and edited the manuscript, and provided funding. Both authors contributed to the article and approved the submitted version.
